# HBeAg Seroconversion in HBeAg-Positive Chronic Hepatitis B Patients Receiving Long-Term Nucleos(t)ide Analog Treatment: A Systematic Review and Network Meta-Analysis

**DOI:** 10.1371/journal.pone.0169444

**Published:** 2017-01-20

**Authors:** Tongjing Xing, Hongtao Xu, Lin Cao, Maocong Ye

**Affiliations:** Department of Infectious Diseases, Taizhou People’s Hospital, Taizhou, Jiangsu Province, China; Universita degli Studi di Pisa, ITALY

## Abstract

**Background:**

HBeAg seroconversion is an important intermediate outcome in HBeAg-positive chronic hepatitis B (CHB) patients. This study aimed to compare the effect of nucleos(t)ide analogs (NAs) on HBeAg seroconversion in treating CHB with lamivudine, adefovir, telbivudine, entecavir, and tenofovir.

**Methods:**

Network meta-analysis of NA treatment-induced HBeAg seroconversion after 1–2 years of treatment was performed. In addition, NA treatment-induced HBeAg seroconversion after 3–5 years of treatment was systematically evaluated.

**Results:**

A total of 31 articles were included in this study. Nine and five studies respectively reporting on 1- and 2-year treatment were included in our network meta-analysis. In addition, 6, 5, and 5 studies, respectively reporting on 3-, 4-, and 5-year treatment were included in our systematic evaluation. Telbivudine showed a significantly higher HBeAg seroconversion rate after a 1 year treatment period compared to the other NAs (odds ratio (OR) = 3.99, 95% CI 0.68–23.6). This was followed by tenofovir (OR = 3.36, 95% CI 0.70–16.75). Telbivudine also showed a higher seroconversion rate compared to the other NAs after a 2 year treatment period, (OR = 1.38, 95% CI 0.92–2.22). This was followed by entecavir (OR = 1.14, 95% CI 0.72–1.72). No significant difference was observed between spontaneous induction and long-term telbivudine treatment-induced HBeAg seroconversion. However, entecavir and tenofovir treatment-induced HBeAg seroconversions were significantly lower than spontaneous seroconversion.

**Conclusion:**

Long-term treatment with potent anti-HBV drugs, especially tenofovir and entecavir, may reduce HBeAg seroconversion compared with spontaneous HBeAg seroconversion rate. Telbivudine treatment, whether short term or long term, is associated with higher HBeAg seroconversion compared with the other NAs. However, the high rates of drug resistance likely limit the application of telbivudine.

## Introduction

Chronic hepatitis B (CHB) naturally involves an alternating process of repeated hepatitis occurrence and disease remission. Interaction and mutual influences between the virus and immune system determines the progression and clinical outcome of CHB [1]. HBeAg, an important antigen expressed by hepatitis B virus (HBV), has been suggested to inhibit the immune function of the host [2]. CHB progression involves either spontaneous or treatment-induced HBeAg seroconversion, with the latter often associated with clinical remission to achieve the inactive virus carrier state. Therefore, HBeAg seroconversion is not only a hallmark of HBeAg-positive CHB patients but also speculated as an important indicator for anti-HBV therapy [3].

Nucleos(t)ide analogs (NAs) are common anti-HBV drugs. Five NAs, namely, lamivudine, adefovir, telbivudine, entecavir, and tenofovir, are currently used to treat CHB [4]. The mechanism of action of NAs involves inhibition of viral replication through direct inhibition of DNA polymerase activity, reducing the reuse of covalently closed circular DNA (cccDNA) and transcription of pregenomic RNA, as well as through indirect effect on HBeAg synthesis. Short-term treatment of HBeAg-positive CHB patients can increase the rate of HBeAg seroconversion. By contrast, long-term treatment increases the histological improvement and regression of fibrosis thus improves the prognosis of CHB patients [5]. However, inhibition of viral replication can disrupt the homeostasis between the virus and immune system of the host, affecting the immune response and probably producing negative effects on HBeAg seroconversion [6].

Entecavir and tenofovir are the most effective anti-HBV drugs, followed by telbivudine and lamivudine, whereas adefovir dipivoxil is the least effective of the five drugs. Entecavir and tenofovir are recommended as first-line therapy for adults with immune-active CHB by the American Association for the Study of Liver Disease, European Association for the Study of the Liver and Asian Pacific Association for the Study of the Liver [4, 7, 8]. However, HBeAg seroconversion rates are different in patients with treatment-naïve HBeAg-positive CHB after 1–2 year of oral NA therapy. The meta-analysis performed by Dakin *et al*. showed that administering more effective anti-HBV drugs does not increase the HBeAg seroconversion rate [9]. The meta-analysis by Wiens *et al*. showed that tenofovir exerts the strongest inducing effect on HBeAg seroconversion after 1 year of treatment [10]. Moreover, no study has systematically evaluated the effect of long-term treatment on HBeAg seroconversion thus far. Herein, we performed a meta-analysis on NA treatment-induced HBeAg seroconversion after 1–2 years of treatment. In addition, NA treatment-induced HBeAg seroconversion after 3–5 years of treatment was systematically evaluated.

## Methods

### Search strategy

The data used in this research were obtained from PubMed (MEDLINE), EMBASE, and Cochrane Library. The following search terms were used: HBeAg-positive CHB and Entecavir (ETV) or lamivudine (LAM) or Telbivudine (LDT) or Tenofovir (TDF) or Adefovir (ADF), HBeAg seroconversion, and randomized controlled trial (RCT). Studies involving special population groups (see below in inclusion and exclusion criteria) and drugs used in combination were not included. All analyses were based on previous published studies, no ethical approval and patient consent are required.

Studies published from 2000 to 2015 were included. Recent publications were also searched manually. Only randomized controlled trials (RCTs) with durations of approximately 1–2 years were included. In addition, open-label and prospective cohort studies with a duration of approximately 3–5 years were included. One year is defined as 48 or 52 weeks. In addition, spontaneous HBeAg seroconversion of long term follow-up was conducted from manual search results.

### Data collection

Data were extracted and evaluated by two independent reviewers. Reviewers resolved discrepancies through discussion. Studies with a Jadad score [11] of lower than 3 points were excluded. HBeAg seroconversion was the unique index evaluated in this study. We extracted data on study characteristics, patient characteristics, intervention details, and HBeAg seroconversion rate.

### Inclusion and exclusion criteria

The inclusion criteria were as follows: (1) HBeAg-positive adult CHB patients; (2) NA-naive patients; (3) NA monotherapy; and (4) drug dosages were those generally recommended by international guidelines. The exclusion criteria were as follows: (1) drug resistance at the beginning of treatment with NAs; (2) other special population groups, such as children, pregnant, and breast-feeding women, and those receiving immune inhibitor therapy; and (3) patients with decompensated liver cirrhosis.

### Statistical analysis

Analyses were performed using Addis software version 1.16.5 (Drug IS.org, Amsterdam, The Netherlands). Network meta-analysis was conducted to compare the odds ratios (ORs) of the treatments and to rank the therapeutic regimens to determine their HBeAg seroconversion rate. In the absence of significant inconsistency, the relative effects of the interventions were analyzed using a consistency model based on a random-effects Bayesian model provided by the ADDIS software. The analysis results are presented as ORs with associated 95% confidence intervals, as well as estimated probabilities for the interventions in descending order. This meta-analysis was performed according to the Preferred Reporting Items for Systematic Reviews and Meta-analysis(PRISM) statement(S1 PRISMA Checklist) [12].

## Results

### Search results and summary of studies

**Fig 1:**
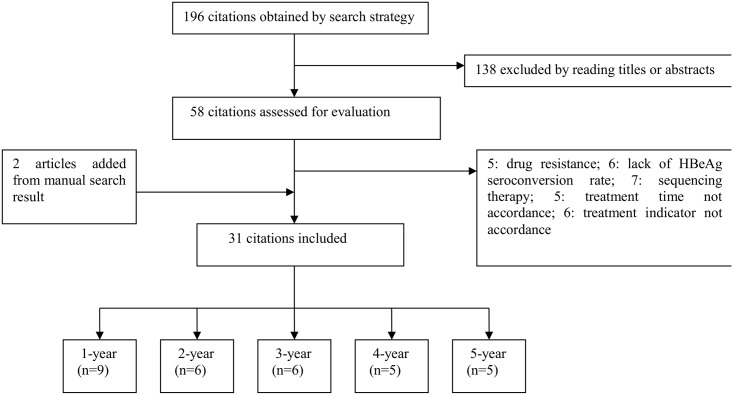
Flow diagram depicting the steps of this systematic review.

In our preliminary search for relevant literature, we identified 196 articles, which included 64 articles from MEDLINE, 120 from EMBASE, and 12 from Cochrane Library. A total of 138 articles were excluded after screening their titles and abstracts. A total of 29 articles were excluded after reading their full text (including conference abstracts from the EMBASE database). Two recently published articles found through manual search were included. Fig 1 describes the details of the selection process. Finally, 31 articles were included in this meta-analysis [13–43]. Nine and six articles respectively reporting on 1- and 2-year treatments were included in our network meta-analysis. In addition, 6, 5, and 5 articles respectively reporting on 3-, 4-, and 5-year treatments were systematically evaluated.

### Characteristics of the included studies

Nine studies involving 3,569 patients and reporting on 1-year treatment met the inclusion criteria for this systematic review and meta-analysis [13–21]. Eight studies were multi-center RCTs, and one study is single-center RCT. Six and three studies were performed within 48 and 52 weeks, respectively (Table 1). Six studies involving 2,417 patients and reporting on 2-year treatment met the inclusion criteria for this systematic review and meta-analysis [22–27]. Five studies were multi-center RCTs, and one was an open-label study (Table 2). Most of the 3–5-year long studies were open-label studies in addition to one RCT and four prospective cohort studies (Tables 3–5) [28–43]. The range of alanine aminotransferase (ALT) was more than 1.0–1.3 upper limit of normal (ULN) and lower than 10 ULN. The lower limit of quantification (LLOQ) of HBV DNA was 20 IU/ml (~100 copies/ml) to 400 copies/ml, whereas that reported in several early studies was 1.6–3.78 pg/ml.

**Table 1:** Characteristics of HBeAg positive chronic hepatitis B patients following 1-year treatment with NAs.

Authors,Year	Country (area)	Patients (N)	Male (N)	Age (Years)	MeanALT (U/L)	MeanHBVDNA	Treatment (Dosage/Day)	Treatmentduration	HBVDNALLOQ	Lower and Upper of ALT	Study design
Chang, *et al*.2006[13]	Taiwan	354	274	35	141±114	9.62±2.01	Entecavir (0.5 mg)	48 w	<300 copies/ml	1.3<ALT<10 ULN	RCT
		355	261	35	146±132	9.69±1.99	lamivudine (100 mg)	48 w	<300 copies/ml	1.3<ALT<10 ULN	
Yao, *et al*. 2006[14]	China	225	185	30	195	8.64	Entecavir (0.5 mg)	48 w	<300 copies/ml	1.3<ALT<10 ULN	RCT
	China	221	184	30	197	8.48	lamivudine (100 mg)	48 w	<300 copies/ml	1.3<ALT<10 ULN	
Hou, *et al*.2008[15]	China	147	118	28	156	9.3	Telbivudine (600 mg)	52 w	<300 copies/ml	1.3<ALT<10 ULN	RCT
	China	143	107	29	157	9.7	lamivudine (100 mg)	52 w	<300copies/ml	1.3<ALT<10 ULN	
Lai, *et al*.2007[16]	Hong Kong	458	333	32	146.4	9.5	Telbivudine (600 mg)	52 w	<300 copies/ml	1.3<ALT<10 ULN	RCT
	Hong Kong	463	351	33	159	9.5	lamivudine (100 mg)	52 w	<300 copies/ml	1.3<ALT<10 ULN	
Marcellin, *et al*. 2008[17]	France	176	119	34	142±102	8.64±1.07	Tenofovir (300 mg)	48 w	<400 copies/ml	ALT>1 ULN	RCT
	France	90	64	34	155±121	8.88±0.93	Adefovir (10 mg)	48 w	<400 copies/ml	ALT>1 ULN	
Sriprayoon, *et al*.2012[18]	Thailand	54	-	42	88.7	6.27±1.81	Tenofovir (300 mg)	48 w	<20 IU/ml	ALT>1 ULN	RCT
		52	-	41	99.9	6.20±1.65	Entecavir (0.5 mg)	48 w	<20 IU/ml	ALT>1 ULN	
Hou, *et al*.2015[19]	China	103	-	-	199±133	8.7±0.87	Tenofovir (300 mg)	48 w	<400 copies/ml	ALT>2 ULN	RCT
		99	-	-	189±122	8.7±0.79	Adefovir (10 mg)	48 w	<400 copies/ml	ALT>2 ULN	
Marcellin, *et al*. 2003[20]	France	171	130	34	139±154	8.25±0.90	Adefovir (10 mg)	48 w	<400 copies/ml	ALT>1 ULN	RCT
		167	71	37	139±131	8.12±0.89	Placebo	48 w		ALT>1 ULN	
Zeng *et a*.*l*, 2006[21]	China	240	201	31	3.9ULN	8.6±1.0	Adefovir (10 mg)	52 w	<300 copies/ml	1.0<ALT<10 ULN	RCT
		120	98	32	3.3ULN	8.5±1.0	Placebo	52 w		1.0<ALT<10 ULN	

LLOQ: Lower limit of quantificationRCT: randomized controlled trials

**Table 2:** Characteristics of HBeAg positive chronic hepatitis B patients following 2-year treatment with NAs.

Authors,Year	Country (area)	Patients (N)	Male (N)	Age (Year)	MeanALT (U/L)	MeanHBVDNA	Treatment (Dosage/Day)	Treatmentduration	HBVDNALLQ	Lower and Upper of ALT	Study design
Gish, *etal*. 2007[22]	Taiwan	354	-	-	-	9.8	Entecavir (0.5 mg)	96 w	<300 copies/ml	1.3<ALT<10 ULN	RCT
		355	-	-	-	9.4	lamivudine (100 mg)	96 w	<300 copies/ml	1.3<ALT<10 ULN	
Yao, *et al*. 2008[23]	China	225	185	30	195	8.64	Entecavir (0.5 mg)	96 w	<300 copies/ml	1.3<ALT<10 ULN	RCT
		221	184	30	197	8.48	lamivudine (100 mg)	96 w	<300 copies/ml	1.3<ALT<10 ULN	
Jia, *et al*.2014[24]	China	147	118	28.1	156.3	9.3	Telbivudine (600 mg)	104 w	<300 copies/ml	1.3<ALT<10 ULN	RCT
		143	107	28.9	156.6	9.6	lamivudine (100 mg)	104 w	<300 copies/ml	1.3<ALT<10 ULN	
Liaw, *et al*.2009 [25]	Taiwan	458	333	32	146.2	9.5	Telbivudine (600 mg)	104 w	<300 copies/ml	1.3<ALT<10 ULN	RCT
		463	351	33	158.9	9.5	lamivudine (100 mg)	104 w	<300 copies/ml	1.3<ALT<10 ULN	
Chen, *et al* 2011[26]	China	32	23	36.8	208.7	7.98	Entecavir (0.5 mg)	96 w	<300 copies/ml		RCT
		33	24	34.2	174.7	7.91	Adefovir (10 mg)	96 w	<300 copies/ml		
Heathcote, *et al*.2008[27]	Canada	176	125	34	142	8.3	Tenofovir (300 mg)	96w	<400 copies/ml	1.0<ALT<10 ULN	Open-label study

**Table 3:** Characteristics of HBeAg positive chronic hepatitis B patients following 3-year treatment with NAs.

Authors,Year	Country (area)	Patients (N)	Male (N)	Age (Year)	MeanALT (U/L)	MeanHBVDNA	Treatment (Dosage/Day)	Treatmentduration	HBVDNALLQ	Lower and Upper of ALT	Study design
Leung, *et al*.2001[28]	Hong Kong	58	42	31	1.7ULN	59.2pg/ml	lamivudine (100 mg)	144 w	1.6 pg /ml	1.0<ALT<10 ULN	Open-label
Lin, *et al*. 2007[29]	China	48	38	27.3	3.0 ULN	8.7	Adefovir (10 mg)	144 w	<300 copies/ml	1.0<ALT<10 ULN	Open-label
Gane, *et al*.2011[30]	New Zealand	213	155	30	112	9.4	Telbivudine (600 mg)	156 w	<300 copies/ml	1.3<ALT<10 ULN	Open-label
Yao, *et al*. 2010[31]	China	160	135	30±9	179±117	8.83±0.86	Entecavir (1.0 mg)	156 w	<300 copies/ml	1.3<ALT<10 ULN	Open-label
Sriprayoon, *et al*.	Thailand	200 (95E+)	-	41.6	68.1	5.91	Entecavir (0.5 mg)	144 w	<20 IU/ml	2.0<ALT<10 ULN	RCT
2015[32]		200 (92E+)	-	41.2	76.8	5.94	Tenofovir (300 mg)	144 w	<20 IU/ml	2.0<ALT<10 ULN	Open-label
Heathcote. *et al*. 2011[33]	Canada	214	183	32.4	117.8	8.86	Tenofovir (300 mg)	144 w	<400 copies/ml	1.0<ALT<10 ULN	Open-label

**Table 4:** Characteristics of HBeAg positive chronic hepatitis B patients following 4-year treatment with NAs.

Authors,Year	Country (area)	Patients (N)	Male (N)	Age (Year)	MeanALT (U/L)	MeanHBVDNA	Treatment (Dosage/Day)	Treatmentduration	HBVDNALLQ	Lower and Upper of ALT	Study design
Chang, *et al*.2004[34]	Taiwan	58	42	31	1.86ULN	50.9pg/ml	lamivudine (100 mg)	192 w	3.78 pg/ml	1.0<ALT<10 ULN	Open-label
Liang, *et al*. 2011[35]	China	95	72	34.7	138.8	5.8	Adefovir (10mg)	192 w	<1000 copies/ml	1.5<ALT<12.5ULN	Prospective cohort study
Wang, *et al*.2013 [36]	China	293	221	29.0	116	9.4	Telbivudine (600 mg)	208 w	<300 copies/ml	1.3<ALT<10 ULN	Open-label
Chen, *et al*.2013[37]	China	30	24	39.0±10.1	266.7±94.2	6.8±0.9	Telbivudine (600 mg)	192 w	<500 copies/ml	-	Prospective cohort study
	China	30	27	37.0±9.8	272.8±98.6	6.7±1.0	Entecavir (0.5 mg)	192 w	<500copies/ml	-	
Heathcote, *et al*. 2011[38]	Canada	130	95	35	138	8.62	Tenofovir (300 mg)	192 w	<400 copies/ml	2.0<ALT<10 ULN	Open-label

**Table 5:** Characteristics of HBeAg positive chronic hepatitis B patients following 5-year treatment with NAs.

Authors,Year	Country (area)	Patients (N)	Male (N)	Age (Year)	MeanALT (U/L)	MeanHBVDNA	Treatment (Dosage/Day)	Treatmentduration	HBVDNALLQ	Lower and Upper of ALT	Study design
Yao, *et al*.2009[39]	China	227	-	-	-	-	lamivudine (100 mg)	240 w	1.6 pg /ml	1.0<ALT<10 ULN	Open-label
Zeng, *et al*.2012[40]	China	240	201	31±9	3.9±3.8ULN	8.64±1.07	Adefovir (10 mg)	240 w	<300 copies/ml	1.0<ALT<10 ULN	Open-label
Zhang, *et al*.2015[41]	China	97	74	33	171	7.7	Telbivudine (600 mg)	240 w	<500 copies/ml	ALT>2 ULN	Prospective cohort study
		99	77	39	102	7.5	Entecavir (0.5 mg)	240 w	<500 copies/ml	ALT>2 ULN	
Chang, *et al*.2010[42]	Taiwan	146	117	36	121.8	9.91	Entecavir (0.5 mg)	240 w	<300 copies/ml	1.3<ALT<10 ULN	Open-label
Marcellin, *et al*.2013[43]	France	164	-	-	-	6.7±1.0	Tenofovir (300 mg)	240 w	<400 copies/ml	ALT>1 ULN	Open-label

### Network meta-analysis of HBeAg-positive CHB patients after 1-year treatment with NAs

**Fig 2:**
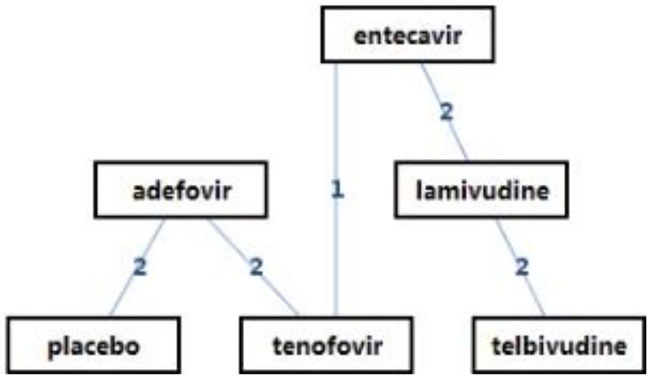
Overview of treatment strategies. Lines represent direct (head-to-head) comparisons.

**Fig 3:**
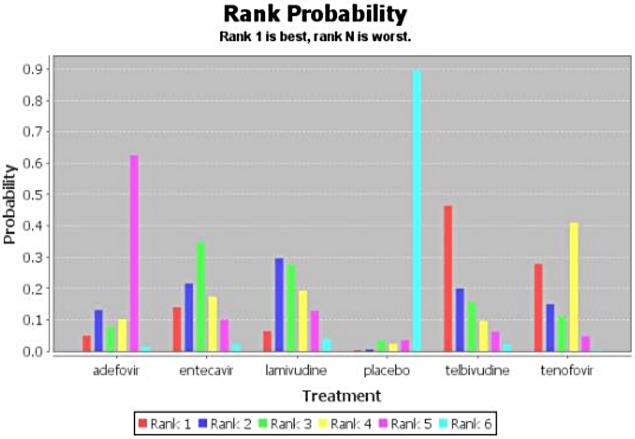
Estimated probabilities for each drug to be ranked for HBeAg seroconversion after 1-year treatment.

Fig 2 shows the network of evidence indicating that 12 possible comparisons could be made, five of which were examined directly in one or more trials. Table 6 shows the ORs of HBeAg seroconversion after 1-year treatment as revealed by network meta-analysis. With regard to HBeAg seroconversion after 1-year treatment, telbivudine achieved a significantly higher rate than the other NAs (OR = 3.99, 95% CI 0.68–23.6) followed by tenofovir (OR = 3.36, 95% CI 0.70–16.75). Placebo achieved the lowest. Fig 3 shows the probabilities of each drug ranked as the choice drug for HBeAg seroconversion.

**Table 6:** Odds ratios of HBeAg seroconversion after 1-year treatment obtained through network meta-analysis.

Network Meta-Analysis (Consistency Model)					
adefovir	1.56(0.39,6.20)	1.53(0.35,6.82)	0.47(0.21,0.99)	1.84(0.38,9.08)	1.46(0.74,3.00)
0.64(0.16,2.57)	entecavir	0.98(0.58,1.72)	0.30(0.06,1.43)	1.17(0.58,2.65)	0.95(0.29,3.08)
0.65(0.15,2.85)	1.02(0.58,1.72)	lamivudine	0.30(0.06,1.64)	1.19(0.72,2.13)	0.97(0.26,3.49)
2.15(1.01,4.82)	3.36(0.70,16.75)	3.31(0.61,17.83)	placebo	3.99(0.68,23.60)	3.15(1.16,9.26)
0.54(0.11,2.60)	0.86(0.38,1.73)	0.84(0.47,1.39)	0.25(0.04,1.46)	telbivudine	0.81(0.19,3.15)
0.68(0.33,1.34)	1.05(0.32,3.46)	1.03(0.29,3.84)	0.32(0.11,0.86)	1.23(0.32,5.18)	tenofovir

### Network meta-analysis of HBeAg-positive CHB patients after a 2-year treatment with NAs

**Fig 4:**
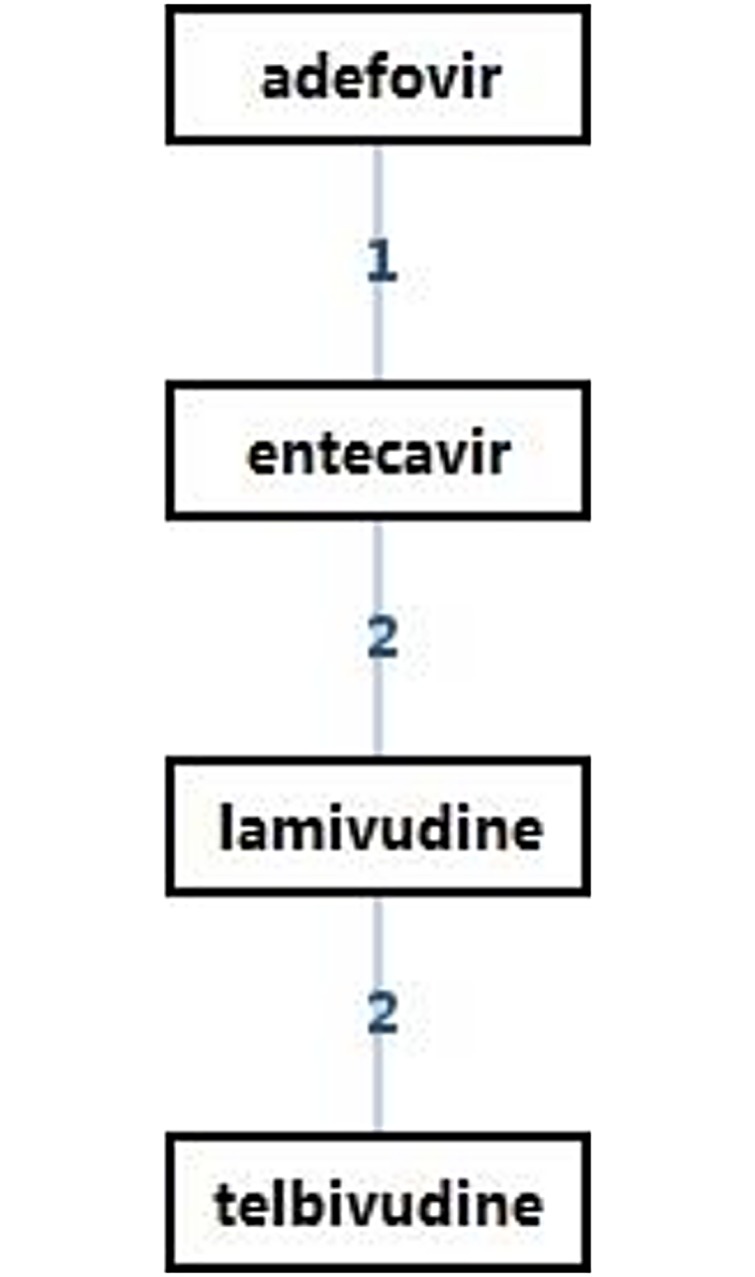
Overview of treatment strategies. Lines represent direct (head-to-head) comparisons.

**Fig 5:**
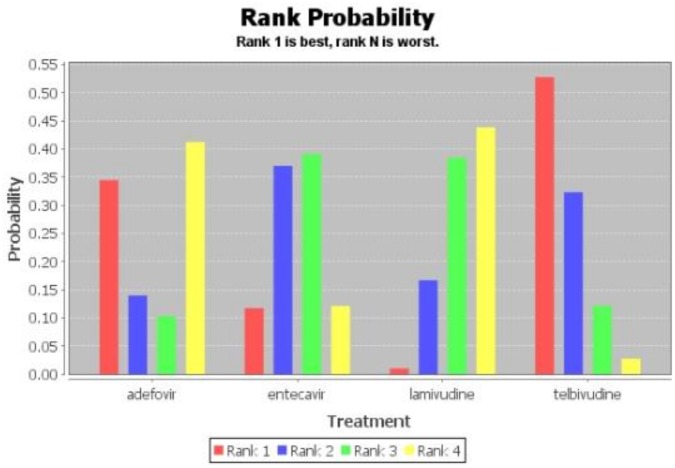
Estimated probabilities for each drug to be ranked as HBeAg seroconversion after a 2-year treatment.

Fig 4 shows the network of evidence indicating that six possible comparisons could be made, four of which were studied directly in one or more trials. Table 7 shows the ORs of HBeAg seroconversion after 2-year treatment as revealed by network meta-analysis. Regarding HBeAg seroconversion after a 2-year treatment, telbivudine achieved a significantly higher rate than the other NAs (OR = 1.38, 95% CI 0.92–2.22) followed by entecavir (OR = 1.14, 95% CI 0.72–1.72). Fig 5 shows the probabilities of each drug to be ranked as the choice drug for HBeAg seroconversion.

**Table 7:** Odds ratios of HBeAg seroconversion after 2-year treatment as revealed by network meta-analysis.

Network Meta-Analysis (Consistency Model)			
adefovir	1.03(0.29, 3.56)	0.90(0.25, 3.52)	1.25(0.32, 5.30)
0.97(0.28, 3.41)	entecavir	0.88(0.58, 1.39)	1.22(0.68, 2.36)
1.11(0.28, 4.08)	1.14(0.72, 1.72)	lamivudine	1.38(0.92, 2.22)
0.80(0.19, 3.11)	0.82(0.42, 1.46)	0.73(0.45, 1.09)	telbivudine

### HBeAg seroconversion of HBeAg-positive CHB patients after 3-, 4-, and 5-year treatments with NAs

Most of the studies were open-label and prospective cohort studies. Table 8 shows the HBeAg seroconversion in HBeAg-positive CHB patients after 3-, 4-, and 5-year treatments with lamivudine, adefovir, telbivudine, entecavir, and tenofovir. Telbivudine was the most effective among the five drugs. In addition, no significant differences were observed between spontaneous seroconversion and telbivudine-induced HBeAg seroconversion. However, HBeAg seroconversion induced by entecavir and tenofovir treatment was significantly lower than spontaneous HBeAg seroconversion.

**Table 8:** Spontaneous and 3-, 4-, and 5-year NA treatment-induced HBeAg seroconversion in HBeAg-positive CHB patients.

Treatment drugs/time	3 years	4 years	5 years	Ref.
Lamivudine	40	47	50	[28, 34, 39]
Adefovir	23.8	41.1	48	[29, 35, 40]
Telbivudine	45.5	53.2	53	[30, 36, 37, 41]
Entecavir	27	30	44	[31, 32, 37, 42]
Tenofovir	26	31	40	[33, 38, 43]
Spontaneous	51.7	59.1	65.2	[50]

## Discussion

Although several studies have shown that short-term NA treatment in CHB patients may improve HBV-specific T cell response [44, 45], NA by itself generally does not exert immune regulation function. Recently, Li *et al*. [46] reported that NA-mediated HBV suppression can downregulate the production of negative regulators of host immunity during the first 24 weeks of therapy and can partially restore the ability of CD8 T cells to secrete pro-inflammatory cytokines. This immune-modulating response may be correlated with the levels of both HBV DNA and HBeAg, but not with NAs. The results of our network meta-analysis suggested that HBeAg seroconversion rates were highest with telbivudine treatment followed by tenofovir therapy. This result is consistent with those from previous studies [47, 48]. The study by Wilens et al. showed that tenofovir was the most effective in inducing HBeAg seroconversion; however, patients receiving combination treatment and who were resistant to drugs were included in that study possibly influencing the accuracy of the results [10]. Given the lack of head-to-head research, the clinical baseline characteristics (e.g., HBV DNA, ALT level, and age) for NA treatment in CHB patients may affect the relative efficacy of drugs. Mealing *et al*. [49] found that when no adjustment was made to account for the differences in baseline viral load among trials, tenofovir was significantly better than entecavir in terms of achieving undetectable viral load after 1-year treatment. However, when they accounted for the impact of baseline viral load, the difference between the two treatments was not significant. ALT levels are positively correlated with the possibility of HBeAg seroconversion. Yuen *et al*. [50] found that the cumulative HBeAg seroconversion rate significantly increased with ALT levels and suggested that high rate of spontaneous HBeAg seroconversion should be considered when treatment for patients with very high ALT levels is initiated.

The effects of long-term NA treatment on immune function of CHB patients remain unclear. Boni *et al*. [51] showed that T cell activity could be restored in patients with suppressed HBV infection following long-term NA treatment *in vitro* despite prolonged exposure to high antigen loads. In this report, when compared with spontaneous induction, HBeAg seroconversion rate was lower in patients who received long-term NA treatment, especially tenofovir and entecavir (40% and 44% after 5-year treatment, respectively). A real world study on the use of lamivudine, adefovir, entecavir, and telbivudine to treat HBeAg-positive CHB patients for 6 years showed that the HBeAg seroconversion rate was 20.0% to 31.6%, although their difference was not statistically significant (X^2^ = 5.81, *P* = 0.214) [52]. These results were significantly lower (41.3%, 47.6%, and 53.5%, after 3-, 4-, and 5-year treatments, respectively) than the reported results for spontaneous induction [50]. Long-term NA treatment is speculated to negatively affect the recovery of immune function among CHB patients. Our results show that the rate of the long-term telbivudine treatment-induced HBeAg seroconversion is relatively higher than the other four NAs. However, the drug resistance rate of telbivudine increases with prolonged treatment time, thereby limiting its application [53]. Kranidioti *et al*. [54] reported that HBV reactivation after withdrawal of NA treatment may contribute to the clearance of HBV associated with disappearance of HBsAg. Most patients who receive anti-HBV NA therapy require more than 10 years to be HBsAg-free, supporting the opposite point of view presented above.

The persistence of HBeAg seroconversion is important for long-term remission of the disease. Spontaneous HBeAg seroconversion may be more stable. The duration of NA treatment-induced HBeAg seroconversion was shorter than that induced by interferon-α [55]. Reijnders *et al*. [56] found that in 132 cases of NA-treated HBeAg-positive CHB patients, the median duration of treatment was 26 months, and 42 cases displayed HBeAg seroconversion. After a median follow-up of 59 months, 13/42 (31%) cases displayed lasting HBeAg seroconversion. Therefore, these results suggested that most HBeAg seroconversion induced by NA therapy is temporary. Long-term treatment regardless of HBeAg seroconversion is necessary. The functionality of T cells with chronic HBV infection is inhibited by the presence of a large number of viral antigens [57]. Application of antiviral drugs to reduce the viral load may thus increase the reactivity of HBV-specific T cells. However, these drugs may also reduce the amount of viral antigens to stimulate immune responses and influence the recovery of immune response function. For instance, in early lamivudine treatment, HBV-specific T cells could be detected, but the recovery of T cell activity was partial and transient, and generally disappeared within approximately 6 months and could not increase the HBeAg seroconversion rate [58]. Long-term NA treatment-induced HBeAg seroconversion rate decreased in this study. Hence, premature intervention with NAs is apparently not conducive for HBeAg seroconversion, and affects the efficiency of treatment to a certain degree for HBeAg-positive CHB patients. This issue needs to be considerated during the clinical practice of HBeAg-positive CHB treatment with NAs, especially for CHB patients with mild disease.

Although this study included RCTs with 1 or 2 years of treatment, long-term effects of these treatments were evaluated with open-label study and cohort studies due to the unethical and infeasibility of withholding long-term treatment in the control group. In the selected RCT, the difference in detection method and lower limits of quantification of HBV DNA may affect the results. The dosages and routes of administration described in specific clinical protocols are different, such as the dosage of entecavir used at the 5 year was 1.0 mg (frequently used 0.5 mg). In addition, the rate of viral resistance was higher in lamivudine and telbivudine used in earlier treatment periods, especially during long-term treatment. This factor may impact the rate of HBeAg seroconversion to a certain extent. HBeAg seroconversion were influenced by the level of ALT, age etc.[59]. Spontaneous HBeAg seroconversion rate was from the a large natural population [50]. The ranges of ALT and age are 1ULN to 6820U/L and 1 to 85 year, respectively. However, the ranges of ALT and age in most of clinical trials are 1.3 ULN to 10ULN and 15 to 65 year, respectively. These differences might affect the comparability of the HBeAg seroconversion rate between the real world study and cilinical trials.

In conclusion, long-term treatment with potent anti-HBV drugs, especially tenofovir and entecavir, may reduce HBeAg seroconversion compared with spontaneous HBeAg seroconversion rate. Telbivudine is associated with higher HBeAg seroconversion compared with the other NAs in both short- or long-term treatment. However, the high rate of drug resistance potentially limits the application of telbivudine.

## Supporting Information

S1 PRISMA Checklist (DOC)
